# The household financial burden of non-communicable diseases in low- and middle-income countries: a systematic review

**DOI:** 10.1186/s12961-021-00732-y

**Published:** 2021-06-21

**Authors:** Joseph Kazibwe, Phuong Bich Tran, Kristi Sidney Annerstedt

**Affiliations:** 1 Department of Global Public Health, Karolinska Institutet, Solna, Sweden; 2 The Health and Social Protection Action Research and Knowledge Sharing Network (SPARKS), Solna, Sweden; 3 Department of Infectious Disease Epidemiology, School of Public Health, Imperial College London, London, UK; 4 Faculty of Medicine and Health Sciences, University of Antwerp, Wilrijk, Belgium

**Keywords:** Noncommunicable diseases, Financial burden, Out-of-pocket expenditure, Catastrophic expenditure, Patient costs, Household costs, Cascade of care, LMICs, Method gaps

## Abstract

**Background:**

The chronic nature of noncommunicable diseases (NCD) and costs associated with long-term care can result in catastrophic health expenditure for the patient and their household pushing them deeper into poverty and entrenching inequality in society. As the full financial burden of NCDs is not known, the objective of this study was to explore existing evidence on the financial burden of NCDs in low- and middle-income countries (LMICs), specifically estimating the cost incurred by patients with NCDs and their households to inform the development of strategies to protect such households from catastrophic expenditure.

**Methods:**

This systematic review followed the PRISMA guidelines, PROSPERO: CRD42019141088. Eligible studies published between 1st January 2000 to 7th May 2020 were systematically searched for in three databases: Medline, Embase and Web of Science. A two-step process, comprising of qualitative synthesis proceeded by quantitative (cost) synthesis, was followed. The mean costs are presented in 2018 USD.

**Findings:**

51 articles were included, out of which 41 were selected for the quantitative cost synthesis. Most of the studies were cross-sectional cost-of-illness studies, of which almost half focused on diabetes and/or conducted in South-East Asia. The average total costs per year to a patient/household in LMICs of COPD, CVD, cancers and diabetes were $7386.71, $6055.99, $3303.81, $1017.05, respectively.

**Conclusion:**

This review highlighted major data and methodological gaps when collecting data on costs of NCDs to households along the cascade of care in LMICs. More empirical data on cost of specific NCDs are needed to identify the diseases and contexts where social protection interventions are needed most. More rigorous and standardised methods of data collection and costing for NCDs should be developed to enable comprehensive and comparable evidence of the economic and financial burden of NCDs to patients and households in LMICs. The available evidence on costs reveals a large financial burden imposed on patients and households in seeking and receiving NCD care and emphasizes the need for adequate and reliable social protection interventions to be implemented alongside Universal Health Coverage.

**Supplementary Information:**

The online version contains supplementary material available at 10.1186/s12961-021-00732-y.

## Background

Non-communicable diseases (NCDs), e.g. diabetes, cancers, cardiovascular diseases and chronic respiratory diseases, persist over an extended amount of time and often cause death after prolonged periods of disability [1, 2]. According to World Health Organization (WHO), NCDs are the greatest cause of mortality worldwide with 41 million deaths accounting for over 70% of total deaths per year [1, 3]. NCDs also lead to 15 million premature deaths per year globally [1, 4] and 85% of premature deaths occur in low- and middle-income countries (LMICs) [3]. The increasing prevalence of NCDs in LMICs [1, 3] in addition to the already existing high burden of communicable diseases is resulting in a dual burden of disease for many countries to manage [2].

Research findings highlighted that the four most prevalent NCDs, along with mental health, would pose accumulative global economic losses of 47 trillion USD by 2030, approximately 75% of global gross domestic product (GDP) [5]. This is anticipated to have disproportionate impacts on LMICs, where health systems are fragile, safety nets are lacking and current efforts to cope with multiple concurrent health issues are ongoing. Over 2 billion people living in LMICs are hindered from an efficient, equitable and adequately funded health care system, in addition to the lack of universal health coverage (UHC) and financial risk protection schemes. Compared to high income countries (HICs), the household financial burden of healthcare in LMICs is much higher where more than 150 million people suffer from catastrophic expenditure every year and unexpected out-of-pocket expenditures for costly services [6]. For example, a study in China found that one stroke episode pushed 37% of patients and their families below the 1 USD per day per capita poverty line and almost two-thirds of uninsured patients were pushed into poverty [7]. In Sudan, a household with a diabetic child spent around a quarter of their income on medical care [8]. In Pakistan, it was found that 63.5% of cancer patients resorted to personal savings while 27% took out loans to pay for care [9].

Indeed, the NCDs pandemic in part originates from poverty and disproportionately affects the poor [10, 11]. Beaglehole et al. outlined the clear associations between social and economic determinants of health and NCDs which left unaddressed, would impede achieving the sustainable development goals (SDGs) [12]. The most vulnerable populations have a higher likelihood of developing and dying prematurely from NCDs due to the limited access to comprehensive services for chronic disease prevention, treatment, and management. In addition, vulnerable populations live in environments where programmes to address chronic diseases are either non-existent or inadequate, which increases their exposure to risk factors of NCDs [13].

While it is evident that NCDs cause financial burden on patients and their households [14–16], the full extent of the burden is unknown across the complex stage-wise process an individual or patient with an NCD follows while seeking and receiving care. This process is referred to as the cascade of care which has typically five stages including prevention, screening and diagnosis, treatment, management, and palliative care. Costs can be incurred at each stage of the cascade involving direct medical and direct non-medical costs as well as indirect costs. Establishing a comprehensive understanding of a patient’s or their household’s cost burden is crucial for policy makers in developing appropriate interventions that mitigate the risk of falling into poverty, particular at which stage of which disease should social protection intervention be prioritized.

Despite the broadening literature on the financial burden caused by NCDs globally, most literature available is disease specific and from high-income countries [17–21]. There is a need to synthesize the costs incurred by patients or their households in LMICs to enable comparisons between different diseases, settings, stages of the care cascade and types of costs.

This study explores existing evidence on the financial burden of the four most prevalent NCDs (diabetes, cancers, cardiovascular disease—CVD and chronic obstructive pulmonary disease—COPD) [22] to households in LMICs, by estimating the costs incurred by NCD patients or their households at the different stages of the cascade of care. This will enable understanding of the complete economic burden of NCDs on patients and their households in LMICs.

Definitions used in this study *Cascade of care:* a stage-wise process, that may be complex but sequential, in which an individual or patient follows while seeking and receiving care for a given condition. There are typically five stages including prevention, screening and diagnosis, treatment, management, and palliative care.

*Perspective:* the point of view adopted during an economic evaluation that identifies which types of costs and health benefits should be included. For example, individual/patient, health system/government or societal perspectives.

*Individual or patient perspective:* includes costs incurred by the patient or the household.

*Health system/government perspective:* looks at the benefits and costs incurred by government or health system.

*Societal perspective:* includes costs incurred by the society.

*Direct costs:* all costs due to resource use attributable to the use of a health care intervention or illness. Direct costs can be split into direct medical costs and direct non-medical costs.

*Direct medical costs:* include the costs of a defined intervention and all follow-up costs for medication and health care interventions in ambulatory, inpatient, and nursing care.

*Direct non-medical costs:* costs incurred in the process of seeking and after receiving health services e. g. transportation/travel costs, food, accommodation, and additional paid caregiver time.

*Indirect costs:* all costs incurred because of cessation or reduction of work productivity as a result of the morbidity and mortality associated with a given disease.

## Methods

This systematic review followed the Preferred Reporting Items for Systematic Reviews and Meta-Analyses (PRISMA) guidelines [23] and a detailed protocol was registered with PROSPERO before data extraction (registration number: PROSPERO 2019 CRD42019141088).

Electronic databases including Medline (Ovid), Embase (embase.com) and Web of Science (Clarivate Analytics) were systematically searched in May 2019, with an additional search in May 2020 for articles published up until 7th May 2020. In order to ensure our findings are comparable to the present-day financial health expenditure paradigm on NCDs, only studies from 2000 onwards were assessed due to changes in health financing policies and the varying costs of healthcare and technology used in NCD care. A number of countries removed user fees (also known as cost sharing, co-financing, or cost-recovery) to improve health service access as well as establish a central budgetary system and health insurance to fund health services [24, 25]. In addition, progress has been made in the deployment of advanced technology used in the diagnosis and treatment of NCDs in LMICs changing the costs of NCD-related health services.

Bibliographies of included articles were reviewed to find relevant articles that fulfilled the inclusion criteria and not previously included. Authors whose abstracts were considered for inclusion, but the full text was not accessible online, were contacted and requested for article access.

A two-step process, qualitative synthesis (step 1) proceeded by quantitative (cost) synthesis (step 2), was followed [26]. The qualitative synthesis describes the studies included in this systematic review, while the quantitative step includes a sub-set of studies which costs that were deemed appropriate to synthesize. Step 1 was necessary to identify the studies that could be synthesized in step 2. The qualitative synthesis included all studies that met the outlined eligibility criteria as described below, while the quantitative (cost) synthesis was a sub-set of these studies and only assessed costs that were similar in how they were collected.

### Eligibility criteria

A study was considered eligible if it reported costs incurred by the patient and or household in seeking and receiving healthcare in LMICs associated with one of the NCDs of interest (i.e. diabetes, cancers, CVD and COPD) and published in English. Grey literature was excluded.

Studies included in the final qualitative synthesis (step 1) explored the extent of the financial burden of NCDs to the patients and or households during at least one of the following stages in the care cascade: prevention, diagnosis, treatment, management, and palliative care. In addition, eligible studies used quantitative methods (e.g. cost-of-illness studies or cost analyses) to collect and estimate costs and were conducted in a LMIC as specified by the World Bank based on gross national income (GNI) per capita [27]. The primary outcome was the cost (both direct and indirect) incurred by the patient or their household in seeking and receiving NCDs care. Studies that scored below five on the Newcastle–Ottawa Quality Assessment Scale (NOS) were excluded due to having a high risk of bias [28, 29]. Detailed inclusion and exclusion criteria are presented in Additional file 2.

### Search strategy

Electronic literature search was conducted in the abovementioned databases. The search strategy involved keywords categorized into four groups: disease (NCDs), setting (LMICs), financial burden (cost/expenditure), and subject (patient, household). Search strategies were tailored to each database in collaboration with a team of medical librarians from Karolinska Institute. Additional file 3 describes the detailed search strategies for each database used.

### Study selection

The PRISMA guidelines [30] were followed in the selection process. The number of articles retrieved were listed and uploaded to Rayyan QCRI software which was used to identify and remove duplicates [31]. Eligibility of identified studies was assessed independently by the joint first authors (PT and JK). A standard protocol of reading the title, abstract, and full text was followed. All conflicts were discussed and agreed upon through consensus with the senior author (KSA) when applicable.

Studies included in the qualitative analysis (step 1) were further assessed independently by PT and JK for eligibility for the quantitative (cost) synthesis (step 2) based on the similarity in method of data collection and presentation.

### Data collection process

A data extraction form including author, year of publication, type of disease, context (location, setting- urban or rural, social economic status), costing perspective, costing year, currency used, payer, source of cost data, costing time frame, direct medical costs, direct non-medical costs, indirect costs, monthly household income was developed in REDCap [32]. It was piloted on 15 randomly selected articles and modifications deemed necessary were made.

**Fig. 1:**
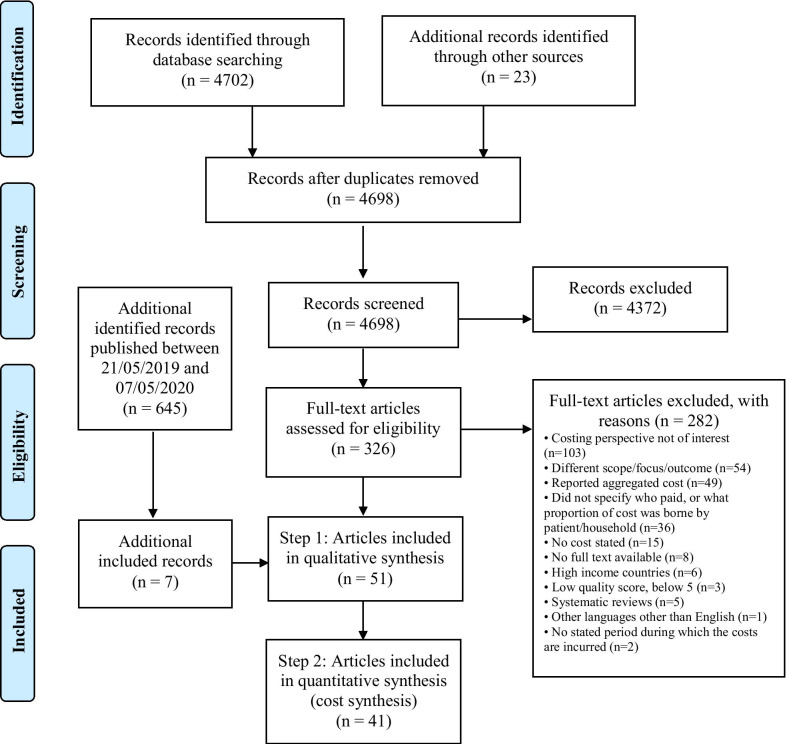
Flow diagram of the study selection process

Simultaneously during the data extraction process, studies were assessed for similarity in cost ingredients to be included in the quantitative (cost) synthesis (Fig. 1). This was performed with a designed checklist of the individual study methods and cost ingredients (Additional file 1).

### Risk of bias in individual studies

Quality of the articles was assessed using the Newcastle–Ottawa Quality Assessment Scale (NOS) [33, 34]. The NOS is a nine-point scale used to assess methodological rigor based on three quality categories: study selection, comparability of study groups, and outcome assessment for most studies types (cohort, cross sectional) and selection, comparability and exposure for case control studies [35]. Any discrepancies were addressed by a joint re-evaluation of the article among all authors.

### Summary measures and synthesis of results (step 2)

The summary measures used in this study were mean and median costs identified during the data extraction. Although the studies included used different costing perspectives (e.g. patient, health system or societal), the costs extracted during the review were costs incurred by patients or the households.

Synthesis of the costs were performed to estimate average costs incurred by type of cost (i.e. direct medical costs, direct nonmedical costs, indirect costs), setting (i.e. low-income countries (LICs), lower-middle income countries, upper-middle income countries (UMICs)) and disease (i.e. diabetes, cancers, CVD and COPD). Average costs were calculated for only costs reported as means. The costs were adjusted for inflation to 2018 using the local consumer price index as reported by the World Bank [36] and costs were converted from the local currency to USD 2018 using the World Bank official average exchange rate [37] when necessary. The geographical location of the study settings was grouped based on WHO Cost Effectiveness and Strategic Planning (WHO-CHOICE) which divided the world into six regions: African Region, Eastern Region, Mediterranean Region, European Region, Region of the Americas, and South-East Asian Region [38].

## Results

### Study selection

After 5343 abstract and title screenings and 362 full-text article reviews, 51 articles met the study inclusion criteria (Fig. 1). The main the reasons for excluding studies were: (1) costing perspective not of interest (*n* = 103); (2) reported different outcome, e.g. cost per capita, cost per visit/admission (*n* = 54); (3) reported aggregated costs (*n* = 49); (4) disaggregated costs presented without specifying the proportion that was paid by the patient or household (*n* = 36); and (5) other reasons (*n* = 40).

### Study characteristics

**Fig. 2:**
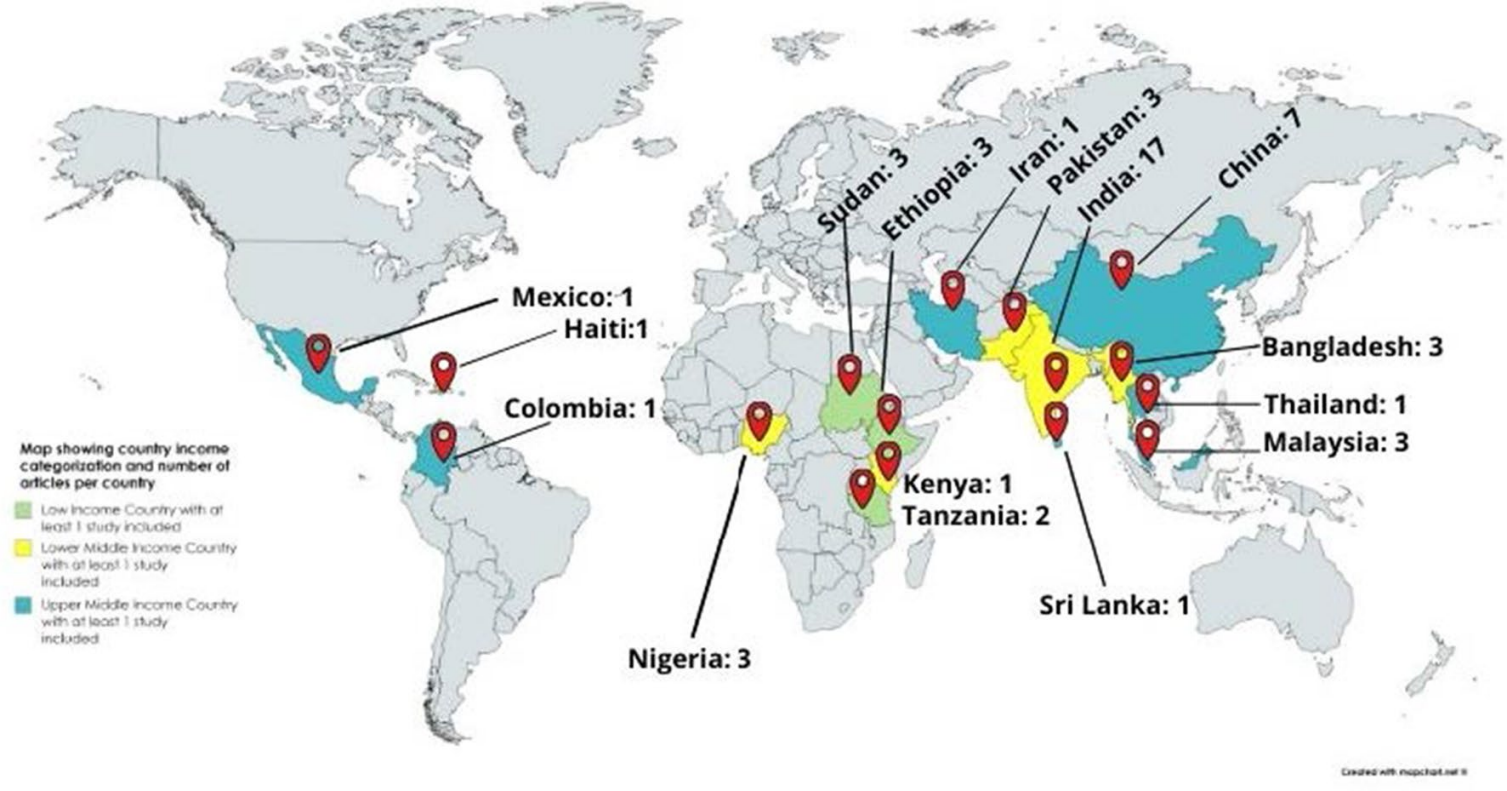
Mapping of selected articles by country *n* = 51

A total of 51 articles were included in the study (Fig. 2). Most studies were conducted in South East Asia (43.1%), out of which the majority were from India [17] and China [7].

The study designs were cross-sectional (78.4%), cohort (19.6%) and case control (2.0%) (Table 1). Most studies were published after 2010 (80.4%) and over half of all included studies were published after 2016. Of the 51 studies, 5 (9.8%) were carried out in low-income countries (LICs) and 30 (58.8%) in lower-middle income countries with over half in India alone.

**Table 1:** Summary characteristics of selected articles, *n* = 51

Summary characteristics of articles	
Study characteristic	Number and percentage of articles *n* (%) *N* = 51
*Type of study*	
Cross sectional	38 (74.5)
Case control	1 (2.0)
Cohort/longitudinal	12 (23.5)
*Year of publication*	
2000–2005	3 (5.9)
2006–2010	7 (13.7)
2011–2015	15 (29.4)
After 2016	26 (51.0)
*Study setting by WHO region*	
Africa	10 (19.6)
Europe	0 (0)
South-East Asia	22 (43.1)
Eastern Mediterranean	6 (11.8)
The Americas	4 (7.8)
Western pacific	9 (17.6)
*World Bank classification of the country*	
Low Income Country	5 (9.8)
Lower-Middle Income Country	30 (58.8)
Upper-Middle Income Country	16 (31.4)
*Source of data*	
Survey	15 (29.4)
Hospital/medical records	27 (52.9)
Both	9 (17.6)
*Cascade of care*^a^	
Prevention	0 (0)
Diagnosis	16 (31.4)
Treatment	31 (60.8)
Disease management	49 (96.1)
Palliative care	9 (17.6)
*Type of costs included*^b^	
Direct medical costs	48 (94.1)
Direct nonmedical costs	38 (74.5)
Indirect costs	21 (41.2)
*Perspective of the study*	
Household/patient only	47 (92.2)
Societal perspective	4 (7.8)
*Diseases*	
Diabetes	22 (43.1)
Cancers	17 (33.3)
CVD	11 (21.6)
COPD	1 (2.0)
*Quality score*	*Mean (*+ */ − SD)*
Cross-sectional studies (*n* = 38)	6.7 (1.34)
Case control studies (*n* = 1)	8 (0)
Cohort studies (*n* = 12)	6.8 (1)

^a^Some studies covered multiple stages in the cascade of care

^b^Some studies included more than one type of costs (i.e. direct medical costs, direct nonmedical costs and indirect costs)

The sources of data varied among studies, most used cost data from hospital/medical records (52.9%), approximately a third used patient cost surveys (29.4%) and few used both (17.6%). Studies generally did not report costs according to specific stages in the cascade of care. The majority of studies (96.1%) estimated the costs incurred during the disease management stage and no study investigated the costs in the prevention stage. Less than a third of the studies collected and analysed costs during the diagnosis (16/51) and palliative care (9/51) level, respectively.

Diabetes was the most studied (43.1%) and COPD was the least studied with only one study included (2.0%). CVD was included in 11 (21.6%) and cancers in 17 (33.3%) articles.

**Fig. 3:**
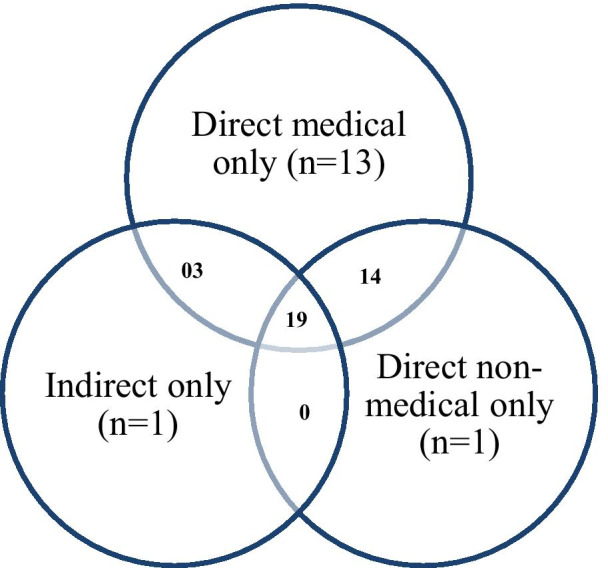
Number of selected articles by types of cost investigated

As described in Fig. 3, the majority of costs captured in the studies were direct medical costs, reported in 49 studies (96.1%); followed by direct non-medical cost from 34 studies (66.7%) and 23 studies (45.1%) reported indirect costs. Most studies captured both direct medical and non-medical costs and almost half of all studies captured all three.

### Quality assessment

The mean NOS score for all the studies included was 6.8. The mean score was lowest among cross-sectional studies (6.7) followed by longitudinal studies with a mean score of 6.8. The highest score [8] was reported among the only case control study included. While the overall quality score was relatively high, many weaknesses were identified including: lack of randomization in study participants, convenience sampling methodology, lack of a comparator/control arm, no explanation regarding the non-response rate and how missing data were addressed, and over 50% of the studies did not validate the self-reported information with potential records.

### Results of individual studies and cost synthesis

A total of 41 studies (80%) were selected for step 2, the quantitative (cost) synthesis, based on similarity in cost ingredients (Additional file 1). It was not possible to analyse the NCD costs at the different cascade of care stages since most studies did not segregate cost by stage. Therefore, the synthesis of NCD costs was presented by disease, country income level and type of cost (Table 2).

**Table 2:** Annual cost (in USD 2018) to a patient/household per disease area by country income categorization (*n* = 41)

Disease group	Country income level	Average annual costs (2018 US dollars)						
		Direct medical costs		Direct nonmedical costs		Indirect costs		Total
		Number of articles included in the analysis	Amount	Number of articles included in the analysis	Amount	Number of articles included in the analysis	Amount	
Diabetes	Upper-middle-income countries	4	505.02	2	10.37	2	89.34	604.73
	Lower-middle-income countries	12	546.93	10	479.91	7	75.97	1102.82
	Low-income countries	0	0.00	0	0.00	0	0.00	0.00
	Total (LMIC)	16	536.46	12	401.66	9	78.94	1017.05
Cancers	Upper-middle-income countries	5	3387.85	4	1042.73	3	333.07	4763.65
	Lower-middle-income countries	9	1051.70	5	475.18	3	276.16	2084.13
	Low-income countries	1	258.72	1	44.49	1	316.67	619.88
	Total (LMIC)	15	2051.02	10	823.91	7	428.87	3303.81
CVD	Upper-middle-income countries	3	720.49	3	1606.59	2	9447.51	11,774.60
	Lower-middle-income countries	2	6230.04	1	1645.66	1	3538.05	11,413.75
	Low-income-countries	4	407.61	4	98.16	4	329.40	835.17
	Total (LMIC)	9	1805.78	8	857.26	7	3392.95	6055.99
COPD	Upper-middle-income countries	0	0.00	0	0.00	0	0.00	0.00
	Lower-middle-income countries	1	5914.11	1	1472.60	0	0.00	7386.71
	Low-income-countries	0	0.00	0	0.00	0	0.00	0.00
	Total (LMIC)	1	5914.11	1	1472.60	0	0.00	7386.71

*LMIC* low- and middle-income countries

Diabetes (*n* = 16) followed by cancers (*n* = 15) represented the most studies. The highest average annual cost for disease management in all LMICs was found for COPD at $7386.71, followed by CVD at $6055.99. The average cost for cancers was $3303.81 and diabetes incurred the lowest cost among the four diseases at $1017.05 per year (Table 2).

The average annual costs (AAC) of each disease is presented below.

#### Diabetes (AAC: $1017.05)

Sixteen studies on diabetes costing were included in the analysis with the majority of the studies (12/16) from lower-middle income countries and four studies from UMICs. No studies from low income countries (LICs) were identified.

The annual direct medical costs incurred by patients/households in lower-middle income countries were similar to UMICs ($546.93 and $505.02). The average annual direct medical cost for diabetes was $536.46. Direct non-medical costs were also substantially higher in lower-middle income countries than UMICs ($479.91 compared to $10.37). The overall annual direct non-medical cost for diabetes was $401.66. In terms of indirect costs, UMICs had slightly higher indirect costs of $89.34 in comparison to $75.97 incurred in lower-middle income countries. The average annual indirect cost incurred by patients in both lower-middle income countries and UMICs due to diabetes was $78.94. Sudan, a lower-middle income country reported the highest average direct medical cost ($1367.67) incurred by patients.

#### Cancers (AAC: $3303.81)

A total of 15 studies were included in the estimation of the average annual cost of cancers: one study from LICs (Ethiopia), nine studies from lower-middle income countries and five studies from UMICs.

The LICs studies reported the lowest direct medical cost of $258.72 per year, followed by lower-middle income countries at $1051.70 and UMICs with the highest direct medical cost of $3387.85 per year. The average annual direct medical cost of cancers for LMICs in general was $2051.02. Direct non-medical costs were highest among UMICs ($1042.73) and lowest in LICs ($44.49). The average direct non-medical cost for all LMICs was $823.91. Over three-quarters of the cancer-related studies (10/13) reported direct non-medical costs while all 15 studies reported direct medical costs. The amount of indirect costs of cancers were similar in all the three country income categories with the UMICs having the highest ($333.07) and the lower-middle income countries having the lowest ($276.16). China, a UMIC reported the highest average direct medical cost ($5601.24) incurred by cancer patients.

#### CVD (AAC: $6055.99)

Nine studies were included in the cost synthesis for CVD relatively evenly distributed among each country income category. The lower-middle income countries had the highest direct medical costs at $6230.04, followed by the UMICs with $720.49 and LICs with the lowest at $407.61. On the other hand, UMICs and lower-middle income countries had similar direct non-medical costs at approximately $1600, while this figure was only $98.16 for LICs.

Indirect costs varied greatly across the different country income categories in the following order: UMICs, lower-middle income countries and LICs. The indirect costs contributed the biggest portion (56%) of the average cost of CVD in LMICs with an amount of $3392.95 out of the total annual cost of $6055.99. The highest average direct medical costs were reported in India ($6230.04).

#### COPD (AAC: $7386.71)

There was only one study selected for the cost estimation of COPD which was from India. The direct medical costs were $5914.11, direct non-medical costs were $1472.60. The indirect cost was not reported.

## Discussion

**Fig. 4:**
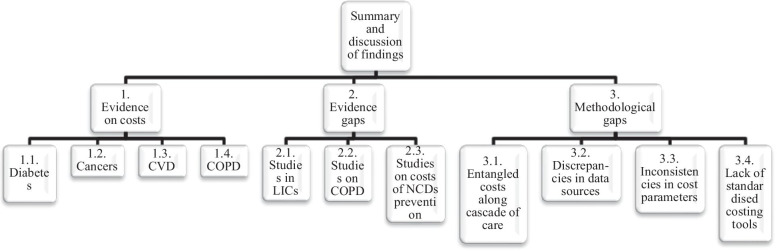
The summary of findings and flow of discussion

To the best of our knowledge, this is the first study that has systematically reviewed this topic with an attempt to disentangle the costs along the cascade of care and according to country income levels. In addition to synthesizing the costs of NCDs, this review has highlighted major gaps in evidence and methodologies (Fig. 4) when assessing the financial burden of NCDs to patients and households along the cascade of care in LMICs. The main findings of this study are that LMICs are incurring high OOP expenditures when seeking care for NCDs and there are several evidence and methodological gaps in knowledge. The household financial burden evidence gaps are mostly in LIC settings, COPD and NCD prevention. The methodological gaps show that studies did not disentangle costs along the cascade of care; discrepancies in data sources, inconsistencies in cost parameters and a lack of standardized tools or protocols to collect costs.

### Available evidence of patient costs of specific NCDs

#### Diabetes

The largest number of studies included in this review was on diabetes. The average direct medical costs of diabetes were slightly lower in UMICs than in lower-middle income countries. This may be due to the variabilities in scopes of insurance benefit, insurance coverage or co-payment across settings. This is in line with the finding that the likelihood of ineffective insurance was lower in UMICs and higher in other lower-middle and LICs [39]. On the contrary, direct non-medical costs in UMICs were substantially higher than that in lower-middle income countries. This may be attributed to the higher costs of living in UMICs [40].

#### Cancers

Cancers yielded the second largest number of studies and studies on cost of cancers were disproportionately conducted in the different income settings: LICs [1], lower-middle income countries [9], UMICs [5].

The direct costs in the LICs, lower-middle income countries and UMICs varied considerably. Notably, the direct medical costs for a patient/household living with cancers in UMICs amounted to over three times that in lower-middle income countries and fourteen times that in LICs. LMICs have retained lower costs of essential cancer drugs by rejecting patent applications. For example, Gleevec, a leukaemia drug that costs $70,000 per year in the United States is $2500 in India [41]. This may explain to some extent the differences in direct medical costs for cancers between LICs/lower-middle income countries and UMICs. In addition, this may also be attributed to a policy to ensure global affordability and access to highly active anti-cancer therapies in higher resource settings [42].

#### CVD

The nine studies included in the calculation of the average annual cost of CVD patients distributed relatively evenly across the three country-income categories.

Based on the finding from the selected studies, the average direct medical costs of CVD to a patient/household in LICs were low compared to that in lower-middle income countries and very low compared to that of UMICs. This could be explained by the fact that in some LICs, CVD services are free of charge at point of use in public and private not-for-profit healthcare centers [43]. Similar to the case of cancers, patients in UMICs incurred much higher direct medical costs than those in lower-middle income countries. This may be the result of new medical technology as a possible driver of increasing costs [44].

#### COPD

There was only one article for COPD that was included in the cost synthesis despite the fact that majority (90%) of COPD deaths occur in LMICs [45].

The study was from a lower-middle income country, specifically India where the direct out-of-pocket spending on COPD was around 5–7 times higher than a study in Mexico [46] and approximately 20 times higher than one from Greece [47]. From this study, COPD has been identified as one of the most expensive NCDs to diagnose and treat. Often, cases are underreported and many go undiagnosed [48]. There still exists, by and large, an absence of patient cost data on COPD globally [45, 49].

### The implications of high costs of NCDs

The chronic nature of NCDs and the high cost associated with long-term care can often result in catastrophic health expenditure for the patients and their household pulling them into or further into poverty [50, 51] and entrenching inequality [52] in society.

Although the costs incurred by households in seeking NCD care is lowest in LICs, it is worth noting that this amount is still considerably high for many people in these countries. For example, the average GDP per capita of LICs as of 2018 was $833 [53] (of which a considerable proportion of people’s annual income in LICs falls below the GDP per capita), and the average costs of cancers in LICs was $619.88 (USD 2018). From this observation alone, it is evident that many people may have been exposed to catastrophic expenditure based on the 40% threshold (i.e. medical cost equal to or exceeding 40% of a household’s income) [54]. In addition to the limited access and availability to NCD care services offered in LICs [55], the high cost associated with NCD care will result in a number of people failing to seek health services for fear of the financial burden. This in turns, drives up incidences of premature mortality due to NCDs. This is consistent to the finding that people living in LICs where health systems are under-funded and financial protections measures for health are insufficient, often struggle the most in paying for health care [56]. With poor access to health services and lack of other forms of financial risk protection in LMICs, the cost of NCDs are often borne by the patient and their relatives [50, 57]. In addition, governments spend almost US$270 per person on health in UMICs and only US$60 per person in lower-middle income countries [58]. Lower government spending on health is inversely proportionate with the higher likelihood of people falling into poverty in seeking health care.

Implementation of UHC in LMICs will provide some financial risk protection against catastrophic expenditures during times of sickness [50, 59–61]. However, UHC alone may not be enough considering the large burden of cost that exists outside the direct medical cost category. Therefore, adequate social protection interventions need to be in place to assist the socioeconomically disadvantaged proportion of the population enabling them to seek NCD care and avoid experiencing catastrophic expenditure along the cascade of care.

### Evidence gaps

#### Few studies in LICs

As indicated above, there are few NCD costing studies performed in LICs yet LICs are known to currently have a considerable proportion of the global NCDs burden that is rapidly increasing. Due to the poor surveillance and disease monitoring systems in LICs, the current estimated burden may be underestimated, and the most affected populations may not be clearly known [62]. Nevertheless, NCDs are known to have a huge financial impact on patients. With the absence of comprehensive data, it may be difficult to estimate the extent of the financial burden and the populations that are critically at risk of catastrophic health expenditure and poverty, thereby hindering the progress of breaking the NCD poverty cycle and achieving the SDGs as described by Beaglehole et al. [12].

#### Scarcity of studies on COPD

Only one costing study of COPD was found eligible, yet COPD is known to be one of the four most prevalent NCDs and one of the most costly diseases to treat/manage [63]. Squire et al. described a similar finding where the majority of published evidence to date relates to tuberculosis (TB) and there is a lack of information for the major non-communicable chronic respiratory diseases: asthma and COPD [64]. Some evidence suggests that the existing tools for measuring, defining, and understanding the full consequences of catastrophic care-seeking costs for these diseases are inadequate; therefore it is proposed that the number and scope of studies of patient costs associated with chronic non-communicable respiratory diseases should be expanded [64].

#### The prevention stage of the cascade of care

Despite the increasing prevalence of NCDs in LMICs and the need to control the incidence of these diseases, none of the included studies explored costs incurred by patients/households to prevent NCDs. Some authors have tried to estimate the cost of prevention of NCDs focusing on secondary prevention but do not specify the costs borne by the patients. Other available studies estimated required resources to prevent NCDs at country and regional level [65]. The lack of cost estimation for the prevention stage may pose a challenge when prioritizing interventions aimed at preventing NCDs in these settings. Although estimating the cost of NCDs prevention by using surveys or clinical trials may be difficult and expensive, modelling studies using real world data can be explored.

### Methodological gaps

#### Entangled costs along the cascade of care

All studies that were included in this review reported costs without disaggregating the costs by stage of care which was similar to the finding of Brouwer et al. [65]. The majority of the studies [66–70] costed only the disease management stage along with aggregating other stages like diagnosis, enrolment into care and palliative care without specifying the costs per stage. With the lack of cost surveillance by stages of care, it is difficult to identify specific cost drivers within each stage and effectively mitigating them with appropriate interventions. Essentially, it creates an obstacle for policymakers to efficiently prioritize interventions and curb catastrophic expenditure at patient/household level.

#### Discrepancies in data sources

We found a clear absence of standardised methodologies for collecting costs, with both costing data sources and practices varying across studies. Some studies relied primarily on self-reported costs—typically collected through the administration of patient questionnaires—while in other studies costs were collected directly from medical records. This latter approach, however, only allowed for the inclusion of direct medical costs, and even then, only those which were incurred within health facilities. Several studies opted for a combination of the two approaches, with some attempting to validate self-reported costs using patients’ receipts [71–73]. While it is clear that self-reported expenditure is better able to capture costs incurred outside health facilities, this approach is perhaps avoided due to the increased risk of bias, with patients often over- or under-reporting (for a range of reasons). It has been shown, however, that these biases can be mitigated through verification with supporting records (e.g. diaries, receipts) [74], supporting the argument that their inclusion should not be overlooked. The evident lack of consistency across methodologies for cost collection, supports the need for standardised frameworks, as previously recognised by Céilleachair et al. [75].

#### Inconsistencies in cost parameters (e.g. cost types and ingredients)

In general, within the scope of each paper, the costs collected were appropriate to answer the designated research question. However, due to the absence of a standardised methodology for collecting cost and the variability of data as mentioned above, it is difficult to assign the most suitable parameters; for example, how exhaustive should the list of cost ingredients be. To mitigate these issues, it is advisable to use the Consolidated Health Economic Evaluation Reporting Standards (CHEERS) guideline when reporting [76].

- *Direct medical costs: *The majority of studies presented direct medical costs, followed by studies that included both direct medical and direct non-medical costs while fewer studies collected indirect costs. This finding is similar to those of Gheorghe et al. [77] which found that most studies on CVD in LMICs included only hospital based costs. Within direct medical costs, the cost items varied widely. For example, some studies collected direct medical costs in aggregated forms of outpatient and inpatient costs [72, 78]; other studies collected costs according to the procedure/service offered to the patient for example, laboratory services/testing, radiotherapy, medicines/drugs, consultation fees, diagnosis among others [73, 73]. The terminology used in the different studies to describe the services received by the patients also varied considerably.
- *Direct non-medical costs: *Some direct non-medical cost ingredients were recurrent among most studies, but the studies were not always consistent in the number of cost ingredients included. For example, some studies costed food and transport only [80, 81] while others further included caretaker cost, and accommodation [79, 82].
- *Indirect costs: *The indirect costs were also calculated differently among the studies. For example, some studies considered the indirect costs to be the value of the lost time when a patient visits the hospital while others included productive time lost by the patient due to sickness, the productive time lost by a household member(s) to take care of the patient and productivity loss due to premature death. The value assigned to the unit time lost was also different as some studies used the average hourly wage rate in a given country while other studies used the exact salary/wage of the patient.

#### Lack of standardised costing tools/protocols (available/used)

There are very few costing tools for NCDs available online. Despite the availability of some costing tools for specific NCDs (e.g. the WHO costing tool for cervical cancer in LMICs [83]), the majority of studies did not use any of these tools.

According to a technical review report[84] commissioned by the WHO, UNICEF, the World Bank and UNFPA on the use of costing tools, it is found that most tools have issues with usability and transparency. The tools are difficult to understand and use without proper training despite having an accompanying user guide. In addition, the computations involved may not be known to the user. The formulas in the tools are usually not clearly stated by the developers resulting in a less user-friendly tool. The rigor and usability of these tools in many cases is not widely tested thereby not being adjustable to suit different contexts or generic quality. In addition, costing tools do not disaggregate costs along the stages of the cascade of care making it impossible to identify the different cost items at the different stages.

This therefore calls for development of disease specific NCD costing tools that will be user friendly and transparent to enable better standardisation and transparency of NCD costing and provide reliable and appraisable costing data that can be used as evidence in informing policy formulation and disease control interventions. As recommended by Brouwer et al. [65] and Céilleachair et al. [75], there is need for more standardised reporting of costs as been undertaken in diseases like TB (85).

### Limitations

Overall, the number of studies found did not capture the costs according to the stages of the cascade of care and therefore it was not possible to present and discuss the costs incurred by the patients and households at the different stages of the cascade. This led to a diversion from the protocol where we had set out to present the financial burden of NCDs by stage of the cascade of care but this was not possible. In addition, due to the limited number of articles of which most were heterogeneous, i.e. diverse costing methods and categorization across different studies, it was difficult to arrive at a more concrete comparison across the different categories of interest (e.g. diseases, income classification, types of cost (medical versus non-medical costs)). Moreover, the cost data was available for a relatively small number of countries; hence, the synthesised cost might not be representative for its disease group and country income level. In order to mitigate this, the cost components were specified in the data extraction template, the cost ingredient checklist and the results section. In many studies self-reported cost data from patient cost surveys was not cross-checked with hospital records/bills and other documentations which could have led to potential over- or under- reporting. In addition, the long costing periods in some of the studies (in which patients were asked to recall the costs they had incurred over a long period of time) may also have created recall bias in cost reporting. There are also lack of controls or comparators in various studies.

Financial and economic burden to households is dependent on the contextual factors of a given territory or country. The amount of resources spent by a household on seeking healthcare depend on the financing mechanism of the health system, the private and public health provider mix, access to health services, strength and reliability of the health system in providing the necessary health services and items needed by a patient. In this study we did not consider the contextual differences that may exist between countries which may impact the cost results. Therefore, the differences within countries of the same World Bank income category may affect the generalizability of the findings.

## Conclusion

This review has shown that there is a lack of evidence on the cost of NCDs to the individual, at different stages of the cascade of care in LMICs. Further research is needed to bridge the evidence and methodological gaps that were identified.

The major evidence gaps identified include: few eligible costing studies that were conducted in LICs or on COPD and there was no eligible study on the costs of NCD prevention. More empirical data on cost of specific NCDs are needed to identify the diseases and contexts where social protection interventions are needed most.

Regarding methodological gaps, studies did not disentangle costs along the cascade of care; there were discrepancies in data sources, inconsistencies in cost parameters and a lack of standardised tools or protocols to collect costs. More rigorous and standardised methods of data collection and costing for NCDs should be developed to enable comprehensive and comparable evidence of the economic and financial burden of NCDs to patients and households in LMICs.

The available evidence on costs reveals a substantial financial burden imposed on patients and households in seeking NCD care and emphasizes the need for adequate and reliable social protection interventions to be implemented alongside UHC. This study may be used by policymakers to inform the development of strategies geared towards protection of NCD patients and their households from catastrophic expenditure by identifying the diseases and contexts where social protection interventions are needed most.

## Supplementary Information

**Additional file 1:** List of included articles and study characteristics; cost ingredient checklist. This table includes all articles that met the inclusion criteria and were selected for review. The table also shows study characteristics as well as the cost ingredient checklist, based on which homogeneity among studies was assessed.

**Additional file 2:** Inclusion and exclusion criteria.

**Additional file 3:** Search strategy.

## Data Availability

The datasets used and/or analysed during the current study are available from the corresponding author on reasonable request.

## Abbreviations

AAC: Average annual costs
COPD: Chronic obstructive pulmonary disease
CVD: Cardiovascular disease
GDP: Gross domestic product
GNI: Gross national income
HICs: High income countries
LICs: Low-income countries
LMICs: Low- and middle-income countries
NCDs: Noncommunicable diseases
NOS: Newcastle–Ottawa Quality Assessment Scale
SGDs: Sustainable development goals
TB: Tuberculosis
UHC: Universal health coverage
UMICs: Upper-middle income countries
UNFPA: United Nations Population Fund
UNICEF: The United Nations Children's Fund
WHO: World Health Organization
