# Supplementary material for: The household financial burden of non-communicable diseases in low- and middle-income countries: a systematic review
Source: Health Res Policy Syst. 2021 Jun 21;19:96. doi: 10.1186/s12961-021-00732-y (PMC8215836; doi:10.1186/s12961-021-00732-y)
Supplement: Supplementary file 2 — Additional file 2: Inclusion and exclusion criteria. [file 12961_2021_732_MOESM2_ESM.docx]

**Additional File 2: Inclusion and Exclusion Criteria**

***Inclusion criteria***

**Types of study to be included**

- Quantitative and mixed methods studies with observational, quasi experimental and experimental study designs are considered for inclusion. The study must include reported costs incurred by a patient or their households in seeking NCD care in LMICs.

**Condition or domain being studied**

- The domain being studied are the costs incurred by a patient with an NCD and their household throughout the cascade of care in LMICs. Costs occur at each stage of the cascade involving direct medical and direct non-medical costs (e.g. transport), indirect costs (e.g. lost income or productivity lost).

**Participants/population**

- Studies with the following participants will be considered; individuals (all ages) with any NCD of interest (diabetes, cancer, cardiovascular diseases and COPD), their households or caregivers in LMICs.

**Costs**

- Studies that state the cost incurred by a patient or household in a given time period with its breakdown

***Exclusion criteria***

- Studies conducted in high income countries
- Qualitative studies
- Systematic reviews
- Studies based on predictive models
- Studies that do not state the costs incurred
- Studies that do not state the period of time during which the costs are incurred
- Studies that states the cost as a cost per visit
- Studies that do not specify who pays costs incurred, or what proportion of cost was borne by the patient/household
- Studies that report costs based on insurance reimbursement data alone
- Studies that report an aggregated cost for NCDs in general
- Studies that report an aggregated cost without cost breakdown (that is direct medical costs, direct none medical costs)
- Studies published in other languages other than English
- Studies published before 1st January 2000
- Studies with no full text available
