# Supplementary material for: The household financial burden of non-communicable diseases in low- and middle-income countries: a systematic review
Source: Health Res Policy Syst. 2021 Jun 21;19:96. doi: 10.1186/s12961-021-00732-y (PMC8215836; doi:10.1186/s12961-021-00732-y)
Supplement: Supplementary file 3 — Additional file 3: Search strategy. [file 12961_2021_732_MOESM3_ESM.docx]

**3. Additional File 3: Search strategy**

3.1. Medline

| Interface: Ovid MEDLINE(R) and Epub Ahead of Print, In-Process & Other Non-Indexed Citations and Daily  Date of Search: 21 May 2019  Number of hits: 2,622  Comment: In Ovid, two or more words are automatically searched as phrases; i.e. no quotation marks are needed | Field labels   - exp/ = exploded MeSH term - / = non exploded MeSH term - .ti,ab,kf. = title, abstract and author keywords - adjx = within x words, regardless of order - * = truncation of word for alternate endings |
| --- | --- |
| 1. exp Chronic Disease/ or (NCD* or chronic disease* or chronic* ill* or chronic condition*).ti,ab,kf. or ((non-communicable or noncommunicable or non communicable or non-infectious or noninfectious or non infectious) adj2 (disease* or illness*)).ti,ab,kf.  2. exp Diabetes Mellitus/ or exp Insulin Resistance/ or (diabet* or insulin*).ti,ab,kf.  3. Cardiovascular Diseases/ or exp Myocardial Infarction/ or exp Coronary Disease/ or exp Heart Failure/ or exp Brain Ischemia/ or exp Stroke/ or Heart Diseases/ or (cardiovascular disease* or heart disease* or cardiac disease* or CVD or myocardial infarct* or heart attack* or cardiovascular stroke* or coronary disease* or coronary artery disease* or coronary heart disease* or coronary arterioscleros* or cardiac failure* or heart failure* or myocardial failure* or brain ischemia* or brain infarct* or cerebral ischemia* or stroke* or cerebrovascular accident* or cerebrovascular event* or vascular event*).ti,ab,kf.  4. exp Breast Neoplasms/ or exp Lung Neoplasms/ or exp Prostatic Neoplasms/ or exp Skin Neoplasms/ or exp Melanoma/ or exp Liver Neoplasms/ or Stomach Neoplasms/ or Urinary Bladder Neoplasms/ or Urologic Neoplasms/ or exp Leukemia/ or exp Multiple Myeloma/ or exp Lymphoma/ or exp Biliary Tract Neoplasms/ or exp Pancreatic Neoplasms/  5. ((breast or mammary or colon* or colorectal* or pulmonary or lung or prostat* or skin* or melanoma* or liver or hepatic* or hepatocellular* or stomach or gastric* or bladder* or urinary tract* or biliary tract* or pancreatic* or pancreas) adj3 (cancer* or carcinoma* or neoplasm* or tumor* or tumour* or malignan*)).ti,ab,kf.  6. (cancer or leukemia* or lymphoma* or myeloma*).ti,ab,kf.  7. exp Lung Diseases/ or (chronic and (obstruct* adj3 (lung* or pulmonar* or airflow*))).ti,ab,kf. or (asthma* or COPD or lung disease* or pulmonary disease*).ti,ab,kf.  8. or/1-7  9. Medically Underserved Area/ or Developing Countries/ or Rural Health/ or Rural Population/  10. (Africa or Asia or Caribbean or West Indies or South America or Latin America or Central America).tw. 11. Afghanistan/ or Albania/ or Algeria/ or Angola/ or Armenia/ or Azerbaijan/ or Bangladesh/ or Benin/ or "Republic of Belarus"/ or Belize/ or Bhutan/ or Bolivia/ or "Bosnia and Herzegovina"/ or Botswana/ or Brazil/ or Bulgaria/ or Burkina Faso/ or Burundi/ or Cambodia/ or Cameroon/ or Cabo Verde/ or Central African Republic/ or Chad/ or exp China/ or Colombia/ or Comoros/ or Congo/ or Costa Rica/ or Cote d'Ivoire/ or Cuba/ or Djibouti/ or Dominican Republic/ or Ecuador/ or Egypt/ or El Salvador/ or Eritrea/ or Ethiopia/ or Fiji/ or Gabon/ or Gambia/ or Georgia/ or Ghana/ or Grenada/ or Guatemala/ or Guinea/ or Guinea-Bissau/ or Guyana/ or Haiti/ or Honduras/ or India/ or Indonesia/ or Iran/ or Iraq/ or Jamaica/ or Jordan/ or Kazakhstan/ or Kenya/ or "Democratic People's Republic of Korea"/ or Kosovo/ or Kyrgyzstan/ or Laos/ or Lebanon/ or Lesotho/ or Liberia/ or Libya/ or Macedonia/ or Madagascar/ or Malaysia/ or Malawi/ or Mali/ or Mauritania/ or Micronesia/ or Mauritius/ or Mexico/ or Moldova/ or Mongolia/ or Montenegro/ or Morocco/ or Mozambique/ or Myanmar/ or Namibia/ or Nepal/ or Nicaragua/ or Niger/ or Nigeria/ or Pakistan/ or "Papua New Guinea"/ or Paraguay/ or Peru/ or Philippines/ or Romania/ or Russia/ or Rwanda/ or Saint Lucia/ or "Saint Vincent and the Grenadines"/ or "Independent State of Samoa"/ or "Sao Tome and Principe"/ or Senegal/ or Serbia/ or Sierra Leone/ or exp Melanesia/ or Sri Lanka/ or Somalia/ or South Sudan/ or Sudan/ or South Africa/ or Suriname/ or Swaziland/ or Syria/ or Tajikistan/ or Tanzania/ or Timor-Leste/ or Thailand/ or Togo/ or Tonga/ or Tunisia/ or Turkey/ or Turkmenistan/ or Uganda/ or Ukraine/ or exp USSR/ or Uzbekistan/ or Vanuatu/ or Venezuela/ or Vietnam/ or Yemen/ or Zambia/ or Zimbabwe/  12. (Afghanistan or Albania or Algeria or Angola or Armenia or Armenian or Azerbaijan or Bangladesh or Benin or Byelarus or Byelorussian or Belarus or Belorussian or Belorussia or Belize or Bhutan or Bolivia or Bosnia or Herzegovina or Hercegovina or Botswana or Brazil or Brasil or Bulgaria or Burkina Faso or Burkina Fasso or Burundi or Urundi or Cambodia or Khmer Republic or Kampuchea or Cameroon or Cameroons or Cameron or Camerons or Cape Verde or Cabo Verde or Central African Republic or Chad or China or Colombia or Comoros or Comoro Islands or Comores or Congo or Costa Rica or Cote d'Ivoire or Ivory Coast or Cuba or Djibouti or Dominica or Dominican Republic or East Timor or East Timur or Timor Leste or Ecuador or Egypt or El Salvador or Eritrea or Ethiopia or Fiji or Gabon or Gambia or Gaza or Georgia Republic or Georgian Republic or Ghana or Grenada or Guatemala or Guinea or Guyana or Haiti or Honduras or India or Indonesia or Iran or Iraq or Jamaica or Jordan or Kazakhstan or Kenya or Kiribati or Democratic People's Republic of Korea or North Korea or Kosovo or Kyrgyz Republic or Lao PDR or Laos or Lebanon or Lesotho or Liberia or Libya or Macedonia or Madagascar or Malaysia or Malaya or Malay or Malawi or Mali or Maldives or Marshall Islands or Mauritania or Mauritius or Mexico or Mehico or Micronesia or Middle East or Moldova or Moldovia or Moldovian or Mongolia or Montenegro or Morocco or Mozambique or Mocambique or Myanmar or Namibia or Nauru or Nepal or Nicaragua or Niger or Nigeria or Pakistan or Palestine or Paraguay or Peru or Philippines or Philipines or Phillipines or Phillippines or Romania or Rumania or Roumania or Russia or Russian or Rwanda or Ruanda or Saint Lucia or St Lucia or Saint Vincent or St Vincent or Grenadines or Samoa or Samoan Islands or Sao Tome or Senegal or Serbia or Sierra Leone or Sri Lanka or Ceylon or Solomon or USSR or Soviet Union or Union of Soviet Socialist Republics Islands or Somalia or South Africa or Sudan or Suriname or Surinam or Swaziland or Eswatini or Syria or Syrian Arab Republic or Tajikistan or Tadzhikistan or Tadjikistan or Tadzhik or Tanzania or Thailand or Togo or Togolese Republic or Tonga or Tunisia or Turkey or Turkmenistan or Tuvalu or Uganda or Ukraine or Uzbekistan or Uzbek or Vanuatu or Venezuela or Vietnam or Viet Nam or West Bank or Yemen or Zambia or Zimbabwe).ti,ab,kf.  13. ((developing or less* developed or under developed or underdeveloped or middle income or low* income or underserved or under served or deprived or poor*) adj3 (countr* or nation? or population? or world or economy or economies)).ti,ab.  14. (low* adj2 (countr* or gdp or gnp or gross domestic or gross national)).ti,ab.  15. (low adj3 middle adj3 countr*).ti,ab.  16. ((rural or remote or nonmetropolitan or underserved or under served or deprived or shortage) adj (communit$ or count$ or area? or region? or province? or district?)).ti,ab.  17. LMIC*.ti,ab,kf.  18. or/9-17  19. "Costs and Cost Analysis"/ or Cost of Illness/ or exp Health Care Costs/ or exp Health Expenditures/ or Economics/ or Economic Status/ or "Fees and Charges"/ or Fees, Medical/ or exp Fees, Pharmaceutical/ or Economics, Medical/ or Medical Indigency/ or Income/ or Sick Leave/ or exp Poverty/ or Working Poor/ or Absenteeism/  20. exp *Neoplasms/ec or exp *Diabetes Mellitus/ec or exp *Chronic Diseases/ec or exp *Cardiovascular Diseases/ec or exp *Lung Diseases/ec  21. ((cost* or expend* or expense* or payment* or spending*) adj3 (catastrop* or direct or health or healthcare or indirect or out-of-pocket)).ti,ab,kf.  22. (cost* adj3 (illness* or sickness or disease*)).ti,ab,kf.  23. (economic* adj3 (condition* or health or healthcare or factor* or medical* or status)).ti,ab,kf.  24. ((economic or financial) adj2 (burden or hardship or stress)).ti,ab,kf.  25. (burden* adj3 (disease* or illness*)).ti,ab,kf.  26. ((cost* or financ* or spending* or payment* or expense* or expend*) adj3 (care or direct service* or drug* or hospital* or medical* or medication* or treatment* or therap*)).ti,ab,kf.  27. (fee* adj2 (dispensing or hospital* or medical* or pharmaceutic* or pharmacy or prescription*)).ti,ab,kf. 28. (affordability or absenteeism or income loss or impoverish* or indigen* or "loss of income" or microeconomic* or poverty or productivity loss* or sick leave).ti,ab,kf.  29. ((sickness or work) adj2 absence).ti,ab,kf.  30. or/19-29  31. Private Sector/ or Family Characteristics/ or Caregivers/ec or exp Financing, Personal/  32. ((caregiver* or home or household* or famil* or individ* or inpatient* or patient* or person* or private or outpatient*) adj4 (bill or bills or budget* or consumption* or cost* or debt* or deficit* or econom* or expend* or expense* or financ* or income* or out-of-pocket or payment* or spending*)).ti,ab,kf.  33. or/31-32  34. 8 and 18 and 30 and 33  35. limit 34 to english  36. remove duplicates from 35 | |

3.2. Embase

| Interface: embase.com  Date of Search: 21 May 2019  Number of hits: 3,201  Comment: Emtree is the controlled vocabulary in Embase | Field labels   - /exp = exploded Emtree term - /de = non exploded Emtree term - ti,ab = title and abstract - NEAR/x = within x words, regardless of order - * = truncation of word for alternate endings |
| --- | --- |
| #1 'chronic disease'/exp OR 'non communicable disease'/de OR ((((ncd*:ti,ab,kw OR chronic:ti,ab,kw) AND disease*:ti,ab,kw OR chronic*:ti,ab,kw) AND ill*:ti,ab,kw OR chronic:ti,ab,kw) AND condition*:ti,ab,kw) OR ((('non communicable' OR noncommunicable OR 'non communicable' OR 'non infectious' OR noninfectious OR 'non infectious') NEAR/2 (disease* OR illness*)):ti,ab,kw) #2 'diabetes mellitus'/exp OR 'insulin resistance'/de OR diabet*:ti,ab,kw OR insulin*:ti,ab,kw #3 'cardiovascular disease'/de OR 'heart infarction'/exp OR 'coronary artery disease'/exp OR 'brain ischemia'/exp OR 'cerebrovascular accident'/exp OR 'heart disease'/exp OR 'cardiovascular disease*':ti,ab,kw OR 'heart disease*':ti,ab,kw OR 'cardiac disease*':ti,ab,kw OR cvd:ti,ab,kw OR 'myocardial infarct*':ti,ab,kw OR 'heart attack*':ti,ab,kw OR 'cardiovascular stroke*':ti,ab,kw OR 'coronary disease*':ti,ab,kw OR 'coronary artery disease*':ti,ab,kw OR 'coronary heart disease*':ti,ab,kw OR 'coronary arterioscleros*':ti,ab,kw OR 'cardiac failure*':ti,ab,kw OR 'heart failure*':ti,ab,kw OR 'myocardial failure*':ti,ab,kw OR 'brain ischemia*':ti,ab,kw OR 'brain infarct*':ti,ab,kw OR 'cerebral ischemia*':ti,ab,kw OR stroke*:ti,ab,kw OR 'cerebrovascular accident*':ti,ab,kw OR 'cerebrovascular event*':ti,ab,kw OR 'vascular event*':ti,ab,kw #4 'lung tumor'/exp OR 'prostate tumor'/exp OR 'breast tumor'/exp OR 'skin tumor'/exp OR 'melanoma'/exp OR 'liver tumor'/exp OR 'stomach tumor'/exp OR 'bladder tumor'/exp OR 'urinary tract tumor'/exp OR 'leukemia'/exp OR 'multiple myeloma'/de OR 'lymphoma'/exp OR 'biliary tract tumor'/exp OR 'pancreas tumor'/exp #5 ((breast OR mammary OR colon* OR colorectal* OR pulmonary OR lung OR prostat* OR skin* OR melanoma* OR liver OR hepatic* OR hepatocellular* OR stomach OR gastric* OR bladder* OR 'urinary tract*' OR 'biliary tract*' OR pancreatic* OR pancreas) NEAR/3 (cancer* OR carcinoma* OR neoplasm* OR tumor* OR tumour* OR malignan*)):ti,ab,kw #6 cancer:ti,ab,kw OR leukemia*:ti,ab,kw OR lymphoma*:ti,ab,kw OR myeloma*:ti,ab,kw #7 'lung disease'/exp OR (chronic:ti,ab,kw AND ((obstruct* NEAR/3 (lung* OR pulmonar* OR airflow*)):ti,ab,kw)) OR asthma*:ti,ab,kw OR copd:ti,ab,kw OR 'lung disease*':ti,ab,kw OR 'pulmonary disease*':ti,ab,kw #8 #1 OR #2 OR #3 OR #4 OR #5 OR #6 OR #7  #9 'afghanistan'/de OR 'albania'/de OR 'algeria'/de OR 'angola'/de OR 'armenia'/de OR 'azerbaijan'/de OR 'bangladesh'/de OR 'benin'/de OR 'republic of belarus'/de OR 'belize'/de OR 'bhutan'/de OR 'bolivia'/de OR 'bosnia and herzegovina'/de OR 'botswana'/de OR 'brazil'/de OR 'bulgaria'/de OR 'burkina faso'/de OR 'burundi'/de OR 'cambodia'/de OR 'cameroon'/de OR 'cabo verde'/de OR 'central african republic'/de OR 'chad'/de OR 'china'/exp OR 'colombia'/de OR 'comoros'/de OR 'congo'/de OR 'costa rica'/de OR 'cote d`ivoire'/de OR 'cuba'/de OR 'djibouti'/de OR 'dominican republic'/de OR 'ecuador'/de OR 'egypt'/de OR 'el salvador'/de OR 'eritrea'/de OR 'ethiopia'/de OR 'fiji'/de OR 'gabon'/de OR 'gambia'/de OR 'georgia'/de OR 'ghana'/de OR 'grenada'/de OR 'guatemala'/de OR 'guinea'/de OR 'guinea-bissau'/de OR 'guyana'/de OR 'haiti'/de OR 'honduras'/de OR 'india'/de OR 'indonesia'/de OR 'iran'/de OR 'iraq'/de OR 'jamaica'/de OR 'jordan'/de OR 'kazakhstan'/de OR 'kenya'/de OR 'north korea'/de OR 'kosovo'/de OR 'kyrgyzstan'/de OR 'laos'/de OR 'lebanon'/de OR 'lesotho'/de OR 'liberia'/de OR 'libya'/de OR 'macedonia' OR 'madagascar'/de OR 'malaysia'/de OR 'malawi'/de OR 'mali'/de OR 'mauritania'/de OR 'micronesia'/de OR 'mauritius'/de OR 'mexico'/de OR 'moldova'/de OR 'mongolia'/de OR 'montenegro'/de OR 'morocco'/de OR 'mozambique'/de OR 'myanmar'/de OR 'namibia'/de OR 'nepal'/de OR 'nicaragua'/de OR 'niger'/de OR 'nigeria'/de OR 'pakistan'/de OR 'papua new guinea'/de OR 'paraguay'/de OR 'peru'/de OR 'philippines'/de OR 'romania'/de OR 'russia'/de OR 'rwanda'/de OR 'saint lucia'/de OR 'saint vincent and the grenadines'/de OR 'independent state of samoa'/de OR 'sao tome and principe'/de OR 'senegal'/de OR 'serbia'/de OR 'sierra leone'/de OR 'melanesia'/exp OR 'sri lanka'/de OR 'somalia'/de OR 'south sudan'/de OR 'sudan'/de OR 'south africa'/de OR 'suriname'/de OR 'swaziland'/de OR 'syria'/de OR 'tajikistan'/de OR 'tanzania'/de OR 'timor-leste'/de OR 'thailand'/de OR 'togo'/de OR 'tonga'/de OR 'tunisia'/de OR 'turkey'/de OR 'turkmenistan'/de OR 'uganda'/de OR 'ukraine'/de OR 'ussr'/exp OR 'uzbekistan'/de OR 'vanuatu'/de OR 'venezuela'/de OR 'vietnam'/de OR 'yemen'/de OR 'zambia'/de OR 'zimbabwe'/de #10 'afghanistan':ti,ab,kw OR 'albania':ti,ab,kw OR 'algeria':ti,ab,kw OR 'angola':ti,ab,kw OR 'armenia':ti,ab,kw OR 'armenian':ti,ab,kw OR 'azerbaijan':ti,ab,kw OR 'bangladesh':ti,ab,kw OR 'benin':ti,ab,kw OR 'byelarus':ti,ab,kw OR 'byelorussian':ti,ab,kw OR 'belarus':ti,ab,kw OR 'belorussian':ti,ab,kw OR 'belorussia':ti,ab,kw OR 'belize':ti,ab,kw OR 'bhutan':ti,ab,kw OR 'bolivia':ti,ab,kw OR 'bosnia':ti,ab,kw OR 'herzegovina':ti,ab,kw OR 'hercegovina':ti,ab,kw OR 'botswana':ti,ab,kw OR 'brazil':ti,ab,kw OR 'brasil':ti,ab,kw OR 'bulgaria':ti,ab,kw OR 'burkina faso':ti,ab,kw OR 'burkina fasso':ti,ab,kw OR 'burundi':ti,ab,kw OR 'urundi':ti,ab,kw OR 'cambodia':ti,ab,kw OR 'khmer republic':ti,ab,kw OR 'kampuchea':ti,ab,kw OR 'cameroon':ti,ab,kw OR 'cameroons':ti,ab,kw OR 'cameron':ti,ab,kw OR 'camerons':ti,ab,kw OR 'cape verde':ti,ab,kw OR 'cabo verde':ti,ab,kw OR 'central african republic':ti,ab,kw OR 'chad':ti,ab,kw OR 'china':ti,ab,kw OR 'colombia':ti,ab,kw OR 'comoros':ti,ab,kw OR 'comoro islands':ti,ab,kw OR 'comores':ti,ab,kw OR 'congo':ti,ab,kw OR 'costa rica':ti,ab,kw OR 'cote d`ivoire':ti,ab,kw OR 'ivory coast':ti,ab,kw OR 'cuba':ti,ab,kw OR 'djibouti':ti,ab,kw OR 'dominica':ti,ab,kw OR 'dominican republic':ti,ab,kw OR 'east timor':ti,ab,kw OR 'east timur':ti,ab,kw OR 'timor leste':ti,ab,kw OR 'ecuador':ti,ab,kw OR 'egypt':ti,ab,kw OR 'el salvador':ti,ab,kw OR 'eritrea':ti,ab,kw OR 'ethiopia':ti,ab,kw OR 'fiji':ti,ab,kw OR 'gabon':ti,ab,kw OR 'gambia':ti,ab,kw OR 'gaza':ti,ab,kw OR 'georgia republic':ti,ab,kw OR 'georgian republic':ti,ab,kw OR 'ghana':ti,ab,kw OR 'grenada':ti,ab,kw OR 'guatemala':ti,ab,kw OR 'guinea':ti,ab,kw OR 'guyana':ti,ab,kw OR 'haiti':ti,ab,kw OR 'honduras':ti,ab,kw OR 'india':ti,ab,kw OR 'indonesia':ti,ab,kw OR 'iran':ti,ab,kw OR 'iraq':ti,ab,kw OR 'jamaica':ti,ab,kw OR 'jordan':ti,ab,kw OR 'kazakhstan':ti,ab,kw OR 'kenya':ti,ab,kw OR 'kiribati':ti,ab,kw OR 'north korea':ti,ab,kw OR 'kosovo':ti,ab,kw OR 'kyrgyz republic':ti,ab,kw OR 'lao pdr':ti,ab,kw OR 'laos':ti,ab,kw OR 'lebanon':ti,ab,kw OR 'lesotho':ti,ab,kw OR 'liberia':ti,ab,kw OR 'libya':ti,ab,kw OR 'macedonia':ti,ab,kw OR 'madagascar':ti,ab,kw OR 'malaysia':ti,ab,kw OR 'malaya':ti,ab,kw OR 'malay':ti,ab,kw OR 'malawi':ti,ab,kw OR 'mali':ti,ab,kw OR 'maldives':ti,ab,kw OR 'marshall islands':ti,ab,kw OR 'mauritania':ti,ab,kw OR 'mauritius':ti,ab,kw OR 'mexico':ti,ab,kw OR 'mehico':ti,ab,kw OR 'micronesia':ti,ab,kw OR 'middle east':ti,ab,kw OR 'moldova':ti,ab,kw OR 'moldovia':ti,ab,kw OR 'moldovian':ti,ab,kw OR 'mongolia':ti,ab,kw OR 'montenegro':ti,ab,kw OR 'morocco':ti,ab,kw OR 'mozambique':ti,ab,kw OR 'mocambique':ti,ab,kw OR 'myanmar':ti,ab,kw OR 'namibia':ti,ab,kw OR 'nauru':ti,ab,kw OR 'nepal':ti,ab,kw OR 'nicaragua':ti,ab,kw OR 'niger':ti,ab,kw OR 'nigeria':ti,ab,kw OR 'pakistan':ti,ab,kw OR 'palestine':ti,ab,kw OR 'paraguay':ti,ab,kw OR 'peru':ti,ab,kw OR 'philippines':ti,ab,kw OR 'philipines':ti,ab,kw OR 'phillipines':ti,ab,kw OR 'phillippines':ti,ab,kw OR 'romania':ti,ab,kw OR 'rumania':ti,ab,kw OR 'roumania':ti,ab,kw OR 'russia':ti,ab,kw OR 'russian':ti,ab,kw OR 'rwanda':ti,ab,kw OR 'ruanda':ti,ab,kw OR 'saint lucia':ti,ab,kw OR 'st lucia':ti,ab,kw OR 'saint vincent':ti,ab,kw OR 'st vincent':ti,ab,kw OR 'grenadines':ti,ab,kw OR 'samoa':ti,ab,kw OR 'samoan islands':ti,ab,kw OR 'sao tome':ti,ab,kw OR 'senegal':ti,ab,kw OR 'serbia':ti,ab,kw OR 'sierra leone':ti,ab,kw OR 'sri lanka':ti,ab,kw OR 'ceylon':ti,ab,kw OR 'solomon':ti,ab,kw OR 'ussr':ti,ab,kw OR 'soviet union':ti,ab,kw OR 'union of soviet socialist republics islands':ti,ab,kw OR 'somalia':ti,ab,kw OR 'south africa':ti,ab,kw OR 'sudan':ti,ab,kw OR 'suriname':ti,ab,kw OR 'surinam':ti,ab,kw OR 'swaziland':ti,ab,kw OR 'eswatini':ti,ab,kw OR 'syria':ti,ab,kw OR 'syrian arab republic':ti,ab,kw OR 'tajikistan':ti,ab,kw OR 'tadzhikistan':ti,ab,kw OR 'tadjikistan':ti,ab,kw OR 'tadzhik':ti,ab,kw OR 'tanzania':ti,ab,kw OR 'thailand':ti,ab,kw OR 'togo':ti,ab,kw OR 'togolese republic':ti,ab,kw OR 'tonga':ti,ab,kw OR 'tunisia':ti,ab,kw OR 'turkey':ti,ab,kw OR 'turkmenistan':ti,ab,kw OR 'tuvalu':ti,ab,kw OR 'uganda':ti,ab,kw OR 'ukraine':ti,ab,kw OR 'uzbekistan':ti,ab,kw OR 'uzbek':ti,ab,kw OR 'vanuatu':ti,ab,kw OR 'venezuela':ti,ab,kw OR 'vietnam':ti,ab,kw OR 'viet nam':ti,ab,kw OR 'west bank':ti,ab,kw OR 'yemen':ti,ab,kw OR 'zambia':ti,ab,kw OR 'zimbabwe':ti,ab,kw #11 'africa':ti,ab,kw OR 'asia':ti,ab,kw OR 'caribbean':ti,ab,kw OR 'west indies':ti,ab,kw OR 'south america':ti,ab,kw OR 'latin america':ti,ab,kw OR 'central america':ti,ab,kw #12 'developing country'/de OR 'rural health care'/exp OR 'rural population'/de #13 ((developing OR 'less* developed' OR 'under developed' OR underdeveloped OR 'middle income' OR 'low* income' OR underserved OR 'under served' OR deprived OR poor*) NEAR/3 (countr* OR nation* OR population* OR world OR economy OR economies)):ti,ab #14 (low* NEAR/2 (countr* OR gdp OR gnp OR 'gross domestic' OR 'gross national')):ti,ab #15 (low NEAR/3 middle NEAR/3 countr*):ti,ab #16 ((rural OR remote OR nonmetropolitan OR underserved OR 'under served' OR deprived OR shortage) NEAR/1 (communit* OR count* OR area* OR region* OR province* OR district*)):ti,ab #17 lmic*:ti,ab,kw #18 #9 OR #10 OR #11 OR #12 OR #13 OR #14 OR #15 OR #16 OR #17  #19 'cost'/de OR 'cost of living'/de OR 'health care cost'/de OR 'drug cost'/de OR 'hospital cost'/de OR 'hospitalization cost'/de OR 'cost of illness'/de OR 'economics'/de OR 'health economics'/de OR 'economic status'/de OR 'fee'/de OR 'medical fee'/exp OR 'income'/de OR 'poverty'/de OR 'working poor'/de OR 'medical leave'/de OR 'absenteeism'/de #20 ((cost* OR expend* OR expense* OR payment* OR spending*) NEAR/3 (catastrop* OR direct OR health OR healthcare OR indirect OR 'out of pocket')):ti,ab,kw #21 (cost* NEAR/3 (illness* OR sickness OR disease*)):ti,ab,kw #22 (economic* NEAR/3 (condition* OR health OR healthcare OR factor* OR medical* OR status)):ti,ab,kw #23 ((economic OR financial) NEAR/2 (burden OR hardship OR stress)):ti,ab,kw #24 (burden* NEAR/3 (disease* OR illness*)):ti,ab,kw #25 ((cost* OR financ* OR spending* OR payment* OR expense* OR expend*) NEAR/3 (care OR 'direct service*' OR drug* OR hospital* OR medical* OR medication* OR treatment* OR therap*)):ti,ab,kw #26 (fee* NEAR/2 (dispensing OR hospital* OR medical* OR pharmaceutic* OR pharmacy OR prescription*)):ti,ab,kw #27 affordability:ti,ab,kw OR absenteeism:ti,ab,kw OR 'income loss':ti,ab,kw OR impoverish*:ti,ab,kw OR indigen*:ti,ab,kw OR 'loss of income':ti,ab,kw OR microeconomic*:ti,ab,kw OR poverty:ti,ab,kw OR 'productivity loss*':ti,ab,kw OR 'sick leave':ti,ab,kw #28 ((sickness OR work) NEAR/2 absence):ti,ab,kw #29 #19 OR #20 OR #21 OR #22 OR #23 OR #24 OR #25 OR #26 OR #27 OR #28  #30 'private sector'/de OR 'household'/de OR 'household economic status'/de OR 'household income'/exp #31 ((caregiver* OR home OR household* OR famil* OR individ* OR inpatient* OR patient* OR person* OR private OR outpatient*) NEAR/4 (bill OR bills OR budget* OR consumption* OR cost* OR debt* OR deficit* OR econom* OR expend* OR expense* OR financ* OR income* OR 'out of pocket' OR payment* OR spending*)):ti,ab,kw #32 #30 OR #31  #33 #8 AND #18 AND #29 AND #32 #34 #8 AND #18 AND #29 AND #32 AND [english]/lim #35 #34 AND ('article'/it OR 'article in press'/it OR 'erratum'/it OR 'review'/it) | |

3.3. Web of Science Core Collection

| Interface: Clarivate Analytics  Date of Search: 21 May 2019  Number of hits: 2,136 | Field labels   - TS/Topic = title, abstract, author keywords and Keywords Plus - NEAR/x = within x words, regardless of order - * = truncation of word for alternate endings   Note: sometimes “quotation marks” are needed for single search terms to avoid automatic term mapping (lemmatization). |
| --- | --- |
| #1 TOPIC: ((NCD* or "chronic disease*" or "chronic* ill*" or "chronic condition*"))  #2 TOPIC: ((("non-communicable" or noncommunicable or "non communicable" or "non-infectious" or noninfectious or "non infectious") NEAR/2 (disease* or illness*)))  #3 TOPIC: ((diabet* or insulin*))  #4 TOPIC: (("cardiovascular disease*" or "heart disease*" or "cardiac disease*" or "CVD" or "myocardial infarct*" or "heart attack*" or "cardiovascular stroke*" or "coronary disease*" or "coronary artery disease*" or "coronary heart disease*" or "coronary arterioscleros*" or "cardiac failure*" or "heart failure*" or "myocardial failure*" or "brain ischemia*" or "brain infarct*" or "cerebral ischemia*" or stroke* or "cerebrovascular accident*" or "cerebrovascular event*" or "vascular event*"))  #5 TOPIC: (((breast or mammary or colon* or colorectal* or pulmonary or lung or prostat* or skin* or melanoma* or liver or hepatic* or hepatocellular* or stomach or gastric* or bladder* or "urinary tract*" or "biliary tract*" or pancreatic* or pancreas) NEAR/3 (cancer* or carcinoma* or neoplasm* or tumor* or tumour* or malignan*)) or (cancer or leukemia* or lymphoma* or myeloma*))  #6 TOPIC: ((chronic and (obstruct* NEAR/3 (lung* or pulmonar* or airflow*))) or (asthma* or COPD or "lung disease*" or "pulmonary disease*"))  #7 #1 or #2 or #3 or #4 or #5 or #6  #8 TOPIC: ("Afghanistan" or "Albania" or "Algeria" or "Angola" or "Armenia" or "Armenian" or "Azerbaijan" or "Bangladesh" or "Benin" or "Byelarus" or "Byelorussian" or "Belarus" or "Belorussian" or "Belorussia" or "Belize" or "Bhutan" or "Bolivia" or "Bosnia" or "Herzegovina" or "Hercegovina" or "Botswana" or "Brazil" or "Brasil" or "Bulgaria" or "Burkina Faso" or "Burkina Fasso" or "Burundi" or "Urundi" or "Cambodia" or "Khmer Republic" or "Kampuchea" or "Cameroon" or "Cameroons" or "Cameron" or "Camerons" or "Cape Verde" or "Cabo Verde" or "Central African Republic" or "Chad" or "China" or "Colombia" or "Comoros" or "Comoro Islands" or "Comores" or "Congo" or "Costa Rica" or "cote d`ivoire" or "Ivory Coast" or "Cuba" or "Djibouti" or "Dominica" or "Dominican Republic" or "East Timor" or "East Timur" or "Timor Leste" or "Ecuador" or "Egypt" or "El Salvador" or "Eritrea" or "Ethiopia" or "Fiji" or "Gabon" or "Gambia" or "Gaza" or "Georgia Republic" or "Georgian Republic" or "Ghana" or "Grenada" or "Guatemala" or "Guinea" or "Guyana" or "Haiti" or "Honduras" or "India" or "Indonesia" or "Iran" or "Iraq" or "Jamaica" or "Jordan" or "Kazakhstan" or "Kenya" or "Kiribati" or "North Korea" or "Kosovo" or "Kyrgyz Republic" or "Lao PDR" or "Laos" or "Lebanon" or "Lesotho" or "Liberia" or "Libya" or "Macedonia" or "Madagascar" or "Malaysia" or "Malaya" or "Malay" or "Malawi" or "Mali" or "Maldives" or "Marshall Islands" or "Mauritania" or "Mauritius" or "Mexico" or "Mehico" or "Micronesia" or "Middle East" or "Moldova" or "Moldovia" or "Moldovian" or "Mongolia" or "Montenegro" or "Morocco" or "Mozambique" or "Mocambique" or "Myanmar" or "Namibia" or "Nauru" or "Nepal" or "Nicaragua" or "Niger" or "Nigeria" or "Pakistan" or "Palestine" or "Paraguay" or "Peru" or "Philippines" or "Philipines" or "Phillipines" or "Phillippines" or "Romania" or "Rumania" or "Roumania" or "Russia" or "Russian" or "Rwanda" or "Ruanda" or "Saint Lucia" or "St Lucia" or "Saint Vincent" or "St Vincent" or "Grenadines" or "Samoa" or "Samoan Islands" or "Sao Tome" or "Senegal" or "Serbia" or "Sierra Leone" or "Sri Lanka" or "Ceylon" or "Solomon" or "USSR" or "Soviet Union" or "Union of Soviet Socialist Republics Islands" or "Somalia" or "South Africa" or "Sudan" or "Suriname" or "Surinam" or "Swaziland" or "Eswatini" or "Syria" or "Syrian Arab Republic" or "Tajikistan" or "Tadzhikistan" or "Tadjikistan" or "Tadzhik" or "Tanzania" or "Thailand" or "Togo" or "Togolese Republic" or "Tonga" or "Tunisia" or "Turkey" or "Turkmenistan" or "Tuvalu" or "Uganda" or "Ukraine" or "Uzbekistan" or "Uzbek" or "Vanuatu" or "Venezuela" or "Vietnam" or "Viet Nam" or "West Bank" or "Yemen" or "Zambia" or "Zimbabwe")  #9 TOPIC: (Africa or Asia or Caribbean or "West Indies" or "South America" or "Latin America" or "Central America")  #10 TOPIC: ((developing or "less* developed" or "under developed" or underdeveloped or "middle income" or "low* income" or underserved or "under served" or deprived or poor*) NEAR/3 (countr* or nation* or population* or world or economy or economies))  #11 TOPIC: (low* NEAR/2 (countr* or gdp or gnp or "gross domestic" or "gross national"))  #12 TOPIC: (low NEAR/3 middle NEAR/3 countr*)  #13 TOPIC: ((rural or remote or nonmetropolitan or underserved or "under served" or deprived or shortage) NEAR/1 (communit* or count* or area* or region* or province* or district*))  #14 TOPIC: LMIC*  #15 #8 or #9 or #10 or #11 or #12 or #13 or #14  #16 TOPIC: ((cost* or expend* or expense* or payment* or spending*) NEAR/3 (catastrop* or direct or health or healthcare or indirect or “out-of-pocket”))  #17 TOPIC: (cost* NEAR/3 (illness* or sickness or disease*))  #18 TOPIC: (economic* NEAR/3 (condition* or health or healthcare or factor* or medical* or status))  #19 TOPIC: ((economic or financial) NEAR/2 (burden or hardship or stress))  #20 TOPIC: (burden* NEAR/3 (disease* or illness*))  #21 TOPIC: ((cost* or financ* or spending* or payment* or expense* or expend*) NEAR/3 (care or “direct service*” or drug* or hospital* or medical* or medication* or treatment* or therap*))  #22 TOPIC: (fee* NEAR/2 (dispensing or hospital* or medical* or pharmaceutic* or pharmacy or prescription*))  #23 TOPIC: (affordability or absenteeism or “income loss” or impoverish* or indigen* or "loss of income" or microeconomic* or poverty or “productivity loss*” or “sick leave”)  #24 TOPIC: ((sickness or work) NEAR/2 absence)  #25 #16 or #17 or #18 or #19 or #20 or #21 or #22 or #23 or #24  #26 TOPIC: ((caregiver* or home or household* or famil* or individ* or inpatient* or patient* or person* or private or outpatient*) NEAR/4 (bill or bills or budget* or consumption* or cost* or debt* or deficit* or econom* or expend* or expense* or financ* or income* or "out-of-pocket" or payment* or spending*))  #27 #7 AND #15 AND #25 AND #26  Refined by: DOCUMENT TYPES: ( ARTICLE OR REVIEW OR EARLY ACCESS ) AND LANGUAGES: ( ENGLISH ) | |
